# Study protocol for Full Speed Ahead: an intervention for improved activities in daily life through Frame Running and Goal directed training

**DOI:** 10.1186/s12887-026-07318-z

**Published:** 2026-07-24

**Authors:** Ann-Christin Eliasson, Kristina Löwing, Emma Hjalmarsson, Cecilia Lidbeck, Agnes Adestedt, Julia Starck, Magnus Aspdahl, Josefin Löwing, Emelie Frisk, Johanna Strand, Annika Ericson, Kristina Andersson, Eva Broström, Ola Kvist, Jessica Norrbom, Ferdinand von Walden

**Affiliations:** 1 Neuropediatric Unit, Department of Women’s and Children’s Health, Karolinska Institutet, Stockholm, 171 76 Sweden; 2 Medical Unit Allied Health Professional, Theme Women’s Health and Allied Health Professionals, Karolinska University Hospital, Stockholm, Sweden; 3 Astrid Lindgren’s Children Hospital, Karolinska University Hospital, Stockholm, Sweden; 4 Department of Radiology, Columbia University, New York, USA; 5 Department of Physiology and Pharmacology, Karolinska Institutet, Stockholm, Sweden

**Keywords:** Cerebral palsy, Mobility, Physical activity, Early intervention, Muscle, Goal directed training and Frame running, Walking, Gait

## Abstract

**Background:**

Early and intensive intervention is crucial for children with cerebral palsy (CP), particularly during the period when gross motor activities typically develop. Despite the prevalence of CP, robust evidence for effective early mobility interventions remains limited. Frame Running, which enables children with limited walking ability to engage in dynamic, high-intensity activities, has emerged as a promising approach, yet research on its effect in young children is currently lacking. The aim of this study is to implement and evaluate the effects of a mobility-focused training program combining Frame Running and goal-directed gross motor training, on mobility in every day life and cardiovascular health in children aged 2–5 years with CP.

**Method:**

The study uses a prospective repeated-measures intervention design with assessments conducted during baseline, intervention, and post-intervention follow-up phases. At least 18 children meeting the inclusion criteria (CP diagnosis, age 2–5 years, GMFCS II–IV) will participate. The design consists of three baseline measurements, a 12-week intervention phase and three post-intervention measurements, across approximately 5–6 months. The program combines home-based training and hospital-based group training, with parents as active trainers and therapists providing coaching. Frame Running and goal-directed training are tailored to individual goals, set collaboratively with families. Outcome measures include the Gross Motor Function Measure (GMFM-66), Functional Mobility Scale (FMS), Paediatric Evaluation of Disability Inventory Computerised Assessment Test (PEDI-CAT), time-based mobility tests: ultrasound assessment of muscle size and morphology, gait analysis, accelerometry (7-day activity monitoring), heart rate monitoring during training, and Goal Attainment Scaling (GAS). Feasibility and fidelity will be investigated by questionnaires.

**Discussion:**

By evaluating the Full Speed Ahead program, this study aims to improve understanding of how an early intensive family-centered mobility intervention may influence developmental trajectories and health in children with CP. If effective, the findings will have significant implications for planning both short- and long-term treatment and services for young children with CP. The prospective repeated-measures intervention design allows for both group-level and individual-level analysis of changes over time, thereby strengthening the evidence base for early, intensive, family-centered mobility interventions.

**Trial registration:**

ClinicalTrial.gov NCTO7374432, Registered November 18 2025.

## Background

Cerebral palsy (CP) results from a spectrum of early-acquired lesions affecting the developing brain. These brain abnormalities or injuries occur at varying time-periods during pregnancy or the neonatal period [1]. Such insults can lead to CP and are associated with a spectrum of functional limitations, which frequently manifest as restrictions in physical activities and participation in daily life, an issue that can be observed even in young children. A child’s ability to play, be physically active, and initiate independent mobility extends beyond simply reaching for a toy or getting to a destination. These abilities are crucial for environmental exploration, which underpins learning and supports physical, social, emotional, and cognitive development [2]. According to the the World Health Organization (WHO), young children should be engaged in at least 60 min of moderate-to-vigorous physical activity every day, while simultaneously minimizing sedentary behaviour, to promote optimal health and developmental outcomes [3]. This highlights the importance of providing even young children with CP opportunities to maximize their abilities, encouraging them to engage in high-intensity physical activity and initiate independent mobility to support health from an early age.

An estimated 17 million people currently live with CP (https://worldcpday.org). A significant proportion of individuals with CP experience reduced walking ability. The Gross Motor Function Classification System (GMFCS) categorizes children into five functional levels: children at GMFCS levels I and II walk independently but may have limitations in speed and coordination, while those at levels III to V typically require assistive technology for mobility. In high-income countries, 25% to 40% of children are classified within GMFCS levels III and IV, indicating substantial restrictions in physical activity [4].

Despite the substantial global population of individuals with CP, there remains lack of robust evidence supporting effective early mobility interventions that leverage the therapeutic window provided by rapid brain development and neuroplasticity in early childhood [5]. Yet, it is known that gross motor activities develop rapidly during the first years of life: children at GMFCS levels III and IV typically achieve about 90% of their gross motor potential by three years of age, while those at levels I and II continue to develop gross motor activities for a few more years [6, 7]. This underscores the critical importance of early intervention during the period of typical developmental progression, when the brain and central nervous system are most sensitive and adaptable [8].

It is also known that non-ambulant children are at the highest risk of developing musculoskeletal pathology, especially hip displacement, skeletal muscle contractures, joint deformity, and pain [9]. For example, reduced skeletal muscle volume is evident as early as 9 months of age [10]. Early intervention for this group of children at a young age, aiming to promote gross motor activities, is likely most effective prior to the development of skeletal muscle contractures, typically observed from 0 to 4 years of age. Therefore, evidence based early intervention programs are warranted.

### Evidence for early mobility training

To date, no studies have specifically targeted interventions aimed at increasing the daily dose of intense physical activity to improve health outcomes in children with CP, two to five years of age. Nevertheless, the literature indicates that early, intensive, and task-oriented mobility interventions can enhance gross motor outcomes in children with CP, especially when these interventions are integrated into daily routines [11, 12]. The implementation of accessible, family-centred programmes is crucial for optimising developmental progress, fostering independence, and promoting social participation [13].

Mobility devices play a pivotal role for children classified at GMFCS levels III–V. Evidence suggests that the provision of appropriate postural and mobility devices can improve emotional well-being, facilitate social interaction, support independent mobility, and reduce caregiver burden [14–16]. Walkers and gait trainers have been identified as valuable tools for supporting upright mobility, facilitating independence, and enabling social interaction with peers [17–19].

A range of mobility aids can support early self-initiated mobility in young children, including adaptive ride-on cars, partial body weight support systems, walkers and gait trainers, orthoses, assisting devices for crawling, and manual or powered mobility devices (Cheng et al. 2025). The process of acquiring and implementing these aids is multifaceted, encompassing initial assessment and prescription, device delivery and fitting, as well as thorough education for both caregivers and children. The expertise of clinicians and service providers is crucial for enable successful treatment, which affect both accessibility and outcomes. However, ongoing challenges—such as restricted access to devices, financial limitations, and inadequate training for families—must be addressed to ensure equitable provision and to optimise developmental outcomes [20].

In this context, Frame Running, previously known as RaceRunning, emerges as a promising addition to the range of mobility aids available for children with CP. This activity enables children with limited walking ability to engage in dynamic, high-intensity activity while promoting social participation and enjoyment. Despite its growing popularity, research on Frame Running remains scarce, even among older children [19]. Further studies are therefore needed to explore the potential of Frame Running as an early intervention to enhance daily physical activity, participation, and overall health outcomes in young children with CP.

### Importance of physical activity

Frame Running is a para-sport originally developed for athletes with moderate to severe difficulties in walking and/or running, but it is now increasingly utilised by children with CP [19]. It is one of the few options that enables physical activity at moderate to high intensity for this population. We have previously shown that regular participation in Frame Running positively influences skeletal muscle growth and cardiorespiratory function. Through Frame Running, children of all ages are able to actively engage and strengthen their musculature and stamina, thereby supporting improvements in both mobility and overall functional ability and cardiovascular health [21].

One of the principal physical benefits of Frame Running is the increased use of the skeletal muscles in the lower limbs. In typically developing children, it is evident that various forms of physical activity require distinct physical capacities [22, 23]. For instance, sprinting demands the development of strong muscles capable of producing rapid, forceful movements (i.e. high power), whereas distance running is more dependent on skeletal muscle endurance and robust cardiorespiratory fitness [23]. The maturation of these abilities is intrinsically linked to the processes of skeletal muscle growth and the way movement is practised throughout childhood. In children with CP, however, the scenario is considerably more complex. Although the underlying brain injury is non-progressive, the musculoskeletal system is gradually influenced by the child’s movement patterns and muscle use [24, 25]. Many children with CP encounter challenges in being sufficiently physically active, which can result in smaller and weaker skeletal muscles when compared to their typically developing peers. Additionally, joint mobility typically decline early in life, with fixed contractures—characterised by stiff and shortened muscles and tendons—frequently developing over time [25].

Current research exploring the determinants of movement ability in children with CP remains limited. Existing studies suggest that factors such as impaired selective motor control, disturbances of perception, reduced muscle strength, spasticity, restricted knee joint range of motion, and poor trunk control may all contribute to difficulties in efficient movement or running speed. However, the interplay between these factors and their relative contributions to overall physical capacity have yet to be fully elucidated. Moreover, other elements—including skeletal muscle mass and the extent of subcutaneous fat, which may influence the body’s capacity for physical work—have seldom been investigated in this population.

These considerations underscore the necessity of providing children with CP with tailored support and adaptive training regimes to promote sustained physical activity. In the absence of regular mobility and strengthening training, children are at increased risk of developing progressive functional limitations over time, potentially compromising both their health and independence.

### Framework for the *Full Speed Ahead* program

The *Full Speed Ahead (FSA)* program is designed for children with CP aged 2–5 years (GMFCS levels II–IV) and their families. The program is grounded in the *F-words* framework developed by Rosenbaum and Gorter [26], which comprises six key concepts—Function, Family, Fitness, Fun, Friends, and Future. This framework emphasizes a strengths-based perspective that focuses on what children *can* do rather than on their limitations. Collectively, these elements promote a holistic and empowering approach that supports improvements in physical health, motor function, and social-emotional development, while also fostering family engagement, peer interaction, and a positive outlook on future participation. A collaborative, family-centered approach underpins the intervention, emphasizing partnership, trust, and shared decision-making between professionals and families. This approach includes mutual goal setting, information exchange, and consistent support aligned with family priorities [13, 27, 28].

A central principle of the FSA program is repetition and massed practice, as skill acquisition is strongly dependent on the amount and quality of practice [29]. Children with typical development, for example, acquire walking skills through countless repetitions of standing, stepping, falling, and retrying, gradually enhancing balance, coordination, and strength [30–32]. Research also show that toddlers take up to 15,000 steps per day as they progress toward independent walking [30]. This developmental process occurs naturally through abundant practice opportunities in enriched, socially interactive environments rather than through explicit instruction. In alignment with this understanding, the present intervention aims to provide an enriched environment that promotes both motor development and social engagement [12].

The intervention consists of two primary components: Frame Running training and goal-directed gross motor training, offering complementary forms of practice. Frame Running aims to enhance mobility, health, and participation among children with CP. It provides an opportunity for children to experience the sensation of speed and movement, experiences that may otherwise be inaccessible due to mobility limitations, while engaging in physical activity that is both enjoyable and health-promoting [33]. This activity fosters enjoyment and a sense of achievement, while simultaneously enhancing cardiovascular fitness and overall well-being. Goal-directed gross motor training supports the acquisition of meaningful everyday activities, including maintaining and changing postures, sitting, standing, and mobility transposed into play in a rich environment [11, 12]. In accordance with family-centered practice, parents identify training goals based on each child’s strengths, priorities, and daily contexts [34]. The goal should be SMART: Specific, Measurable, Attainable, Relevant and Time-bound. They play an important role in engaging children to practice. While SMART formulations and structured scaling can support clarity and follow-up, an overly narrow focus on specific and time-bound targets may also have unintended negative effects, such as perceptions of failure and stress [35]. To reduce these risks, goal setting and scaling will be undertaken collaboratively with families, emphasizing strengths and realistic expectations during the intervention period.

The learning process will be facilitated by using the Goal Attainment Scaling (GAS), a five graded scale where each step will be well-defined and possible to evaluate [36].

To promote sufficient intensity and practice dosage, training is implemented across two complementary settings: home-based training and hospital-based group sessions. Parents play an active and central role in both contexts. During group sessions, trainers integrate all elements of the F-words framework—fostering social relationships, enhancing fitness, promoting enjoyment, engaging families, and cultivating optimism for future participation [26]. Group-based activities also enable children and families to learn from peers and experience the motivational benefits of social interaction, which enhances engagement and supports the long-term sustainability of physical activity participation. Although the intensity achieved through this combined approach can be questioned, it is supported by previous studies using similar designs [37, 38]. There is also evidence that, when appropriately supported, parents can deliver high-frequency, intensive training in the child’s natural environment, consistent with the principles of family-centered practice [39, 40]. Overall, this approach requires a substantial commitment from families, particularly given the young age of the children. However, embedding training within everyday routines is expected to facilitate a high number of repetitions and thereby provide an intensity of practice sufficient to effect the motor outcomes.

Coaching and collaboration with parents will have a prominent role in the FSA program. Coaching has a long established tradition in many early learning programs for children with disability [41, 42] and there are some moderate evidence for improving motor outcome in children with CP when a motor learning approach is used [43]. There is no generally accepted definition of coaching, only various definitions ranging from relationship-directed to intervener-directed concepts without a clear distinction between the instructing and coaching of parents [41]. In this study, the trainer will both act as a coach and supervisor for the parents, using an intervention-directed approach. Trainers are expected to be creative, engaging, and responsive to the children’s cues to maintain motivation and enjoyment, providing positive reinforcement throughout the sessions. They will also use structured coaching strategies, especially important for goal-directed gross motor training – such as modelling and demonstration, brief instruction, guided practice when needed, prompting and fading and immediate feedback linked to goal criteria – to support parents in progressing the child toward the pre-set goals.

## Methods

### Ethics and dissemination

Full ethical approval for this study has been obtained from the Swedish Ethical Review Authority (Dnr 2024-05809-01). All parents will receive both oral and written information about the study before signing an informed consent form. Data will be managed in accordance with regulations from the Karolinska University Hospital, including data de-identification, confidentiality and security. The principal investigators (PIs) will be ultimately responsible for communication of any significant protocol changes to the funder, Swedish Ethical Review Authority and study participants in a timely manner. A trial registration is available: ClinicalTrials.gov Identifier 2024-05809-01, Registered November 18 2025. Final study results will be presented at national and international meetings and published in peer-reviewed international journals.

### Hypothesis, primary and secondary objectives

**Fig. 1:**
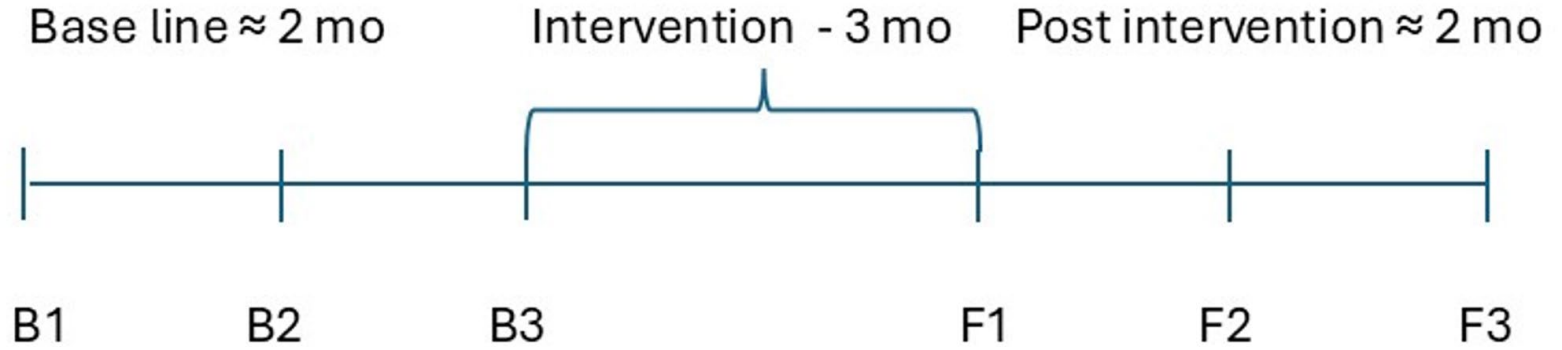
Timeline for the Full Speed Ahead program with prospective repeated-measures intervention design Baseline, and Post intervention

The hypothesis is that mobility performance (e.g., distance, speed, or task-specific scores) will improve during the intervention phase and remain stable or continue to improve during the post-intervention follow-up period.(Fig. 1) Furthermore, we hypothesize that the heart rate–raising activities will lead to improved cardiovascular fitness and skeletal muscle development. The assessments chosen are based on these objectives.

The primary objective is to improve the children’s mobility, with or without assistive devices, through a combination of Frame Running and goal directed gross motor training. The secondary objective is to increase their overall physical activity level through Frame Running.

### The study setting

Astrid Lindgren Children’s Hospital, a tertiary hospital in Stockholm, Sweden.

### Trial design

The study uses a prospective repeated-measures intervention design with assessments conducted during baseline, intervention, and post-intervention follow-up phases. There will be three baseline measurements, a 12-week intervention phase, and three post-intervention measurements (Fig. 1 and Table 1). Primary clinical outcomes are assessed during the baseline and follow-up phases, while process-related measures, see Table 1. The total study period is approximately 5–6 months.

**Table 1:** Assessments to be collected during the training period, B=Baseline, F=Follow up

Assessments	B1	B2	B3	F1	F2	F3
GMFM-66	X	X	X	X	X	X
Funtional Mobility Scale	X	X	X	X	X	X
Time Up and Go	X	X	X	X	X	X
pROM and tonus/MAS		X		X		
Walking/running, 10 m	X	X	X	X	X	X
Frame Running 30 m	X	X	X	X	X	X
Ultra sound	X		X	X		
Gait analysis		X		X		
Pedi-cat, mobility	X	X	X	X	X	X
Prime P/SP	X		X	X		
Accelerometry	X		X	X		X
Goa Attainment Scale			X	X		X
Parent Evaluation						X

This design is informed by principles from single-subject research designs, particularly the use of repeated baseline measurements to characterize within-participant variability before the intervention. However, we do not expect children to return to baseline when the intervention is withdrawn. The planned sample size of at least 18 participants allows also for both individual-level and group-level analyses. This is an advantage when working with heterogeneous populations, such as children with CP, where variability in the magnitude and direction of response to treatment may obscure results in randomized controlled trials (RCTs) [44]. Repeated measurements across phases also allow exploration of individual response patterns and to investigate whether participant characteristics are associated with differential responses to the intervention. Although participants and therapists cannot be blinded to the intervention, outcome assessments are conducted by independent assessors with no involvement in the training phase.

### Recruitment of participants

Children will be recruited through the Astrid Lindgren Children’s Hospital and in collaboration with local habilitation services in Stockholm, Sweden. Eligible participants must meet the following inclusion criteria: a diagnosis of cerebral palsy; age 2–5 years; GMFCS level II–IV; medically stable conditions; and sufficient cognitive ability to benefit from group sessions and follow simple instructions. Both parents and children must have adequate proficiency in Swedish or English as these language skills are essential for participation in data collection and group training. Additionally, families must be able to transport the Frame Runner to training sessions.

Parents of eligible children will first receive verbal information about the study from a senior therapist who is not involved in the child´s regular treatment. Written information will subsequently be provided. Families will then have the opportunity to discuss the study in detail with the investigators before giving informed consent. Families who do not wish to participate will continue to receive usual care through their habilitation services. Eligible families will be identified by clinical staff until the target sample size is reached. This constitutes a convenience sample.

### Statistical method

Demographic and clinical characteristics of the participating children and parents will be summarized using descriptive statistics. Continuous variables will be presented as means and standard deviations or medians and interquartile ranges, as appropriate, while categorical variables will be presented as frequencies and percentages.

Outcome data will be analyzed at both the individual and group levels. At the individual level, graphical displays will be used to visualize changes over time for each participant across study phases. Visual inspection will focus on changes in level, trend, and variability between baseline and follow-up measurements. These graphical analyses will be used to complement the quantitative analyses and to explore individual response patterns. At the group level, outcome measures obtained at baseline and post-intervention follow-up will be compared using appropriate repeated-measures statistical methods. Depending on the distribution and characteristics of the data, analyses may include paired t-tests or non-parametric alternatives such as the Wilcoxon signed-rank test. Effect sizes (e.g., Cohen’s d or equivalent non-parametric effect size measures) will be calculated and reported to facilitate interpretation of the magnitude of change. Given the limited number of observations within each phase, formal assessment of autocorrelation is not expected to provide reliable estimates. Results will therefore be interpreted with consideration of the dependency inherent in repeated measurements obtained from the same participant and in conjunction with graphical inspection of individual trajectories. In cases where graphical and statistical findings diverge, results will be interpreted cautiously.

Missing data will be described for each outcome measure and study phase. The number and proportion of missing observations will be reported together with reasons for missingness when available. Given the limited number of repeated measurements within each phase, missing values will not be imputed. Analyses will be based on available data, and the potential impact of missing observations on the interpretation of findings will be considered when reporting the results. Participant withdrawal and missed assessments will be reported separately.

To ensure for not blinded assessors of GMFM, we have incorporated independent, blinded secondary scoring for a random subset of 20% of the video recordings. Inter-rater reliability (IRR) will be formally assessed using Cohen’s Kappa / ICC. To proactively mitigate bias, if the initial IRR coefficients fall below [e.g., 0.80], a fully blinded primary assessor will be utilized to re-score the entire dataset.

### Sample size

The group-based intervention will include six children per group, with a total of three groups planned. We aim to include at least 18 children. This sample will allow description of response patterns at the individual level as well as analyses at the group level. Repeated measurements across study phases will make it possible to examine changes over time within participants and across the cohort, thereby strengthening the robustness and generalizability of the conclusions regarding intervention effectiveness.

### Training protocol

#### Preparation for the FSA program

The intervention consists of a 12-week combined training programme comprising daily parent-delivered home-based goal-directed training and Frame Running, in addition to a 90-minute group training session held once weekly at Astrid Lindgren Children’s Hospital and supervised by experienced physiotherapists and occupational therapists.

#### Frame runners

Each child will receive their own Frame Runner to be used both at home and during group training. A Frame Runner is a three-wheeled running frame designed to support people with physical disabilities. It has two wheels in the back and one in the front, a saddle that supports the body weight of the user without restricting leg movement, and handlebars that help with steering and stability. The chest plate offers help with stability, and the trunk can be secured with straps if needed. The Frame Runner has no pedals, instead, users propel themselves by running or walking with their feet on the ground, or by using a “leg-swing” pattern in which both legs push off simultaneously. It is available in different sizes and can be used by children as well as adults, allowing them to move independently and at higher speeds than they typically can without support.

At the first baseline assessment, each child will be fitted with a Frame Runner to ensure the correct size, height and proper adjustments. Each child will receive one individually fitted Frame Runner, which they will keep and use throughout the study. The Frame Runner will be stored at the hospital during the baseline phase but will be taken home at the start of the training phase and throughout the training period. Further adjustments will be made when needed. This session will also provide time to become familiar with the Frame Runner and acquire basic experience.

#### Goal setting procedure

The initial goal-setting discussion will start at the second baseline assessment, where the concept of goals will be introduced. This discussion will occur after administration the PEDI-CAT mobility dimension, which helps identify each child’s current functional abilities and highlights areas for further development. The aims should be activity-based and related to mobility (e.g., sit-to-stand, walking, jumping and Frame Running). Families will be actively involved throughout the process, beginning with an exploration of the child’s interests. The discussion will focus on meaningful, realistic activities in the child’s daily environment that can be integrated into daily routines to support intensity of training. Parents will be encouraged to go home and reflect on the potential goals or alternative goals that are realistic and attainable within a three-month period. At the third baseline assessment, two to three individualized goals will be discussed and finalized for each child. The procedure will include describing baseline performance and specifying intermediate steps leading toward achieving the final goals. Goals achievement will be carefully aligned with each child’s current level, ensuring that they are challenging yet attainable. The scaling will be reviewed with the PIs to ensure one-dimensionality.

Once the goal is finalized, the family and the therapist will discuss strategies for integrating them into daily routines. For each goal, a Goal Attainment Scaling (GAS) will be applied, specifying the present level, the expected level of performance and criteria for exceeding expectation (see assessments). A completed form will be provided for the family. Each specific goal will also be recorded by the families in the child’s daily environment.

### Procedure for weekly group training

The weekly group training session, lasting 90 min, will be conducted at Astrid Lindgren Children’s Hospital, Stockholm, Sweden. The intervention will take place in an open indoor facility divided into two areas: one for Frame Running and one for goal directed gross-motor training. A team of one occupational therapist and three physiotherapists will lead the sessions. The therapists responsible for group-based activity training are all very experienced in pediatrics, with a special interest in intensive training and physical activities. Parents act as the primary trainers for their child while therapists will facilitate and coach, supporting parents as a guide for their child through the activities.

The training session will begin with a short group gathering designed to establish routine, engagement and connection. This will include a welcome song, introduction of children, parents and therapists as well as relevant information. The children will then be divided into two subgroups: one will start with 30 min of Frame Running, and the other with 30 min of goal directed gross-motor training. During both activity sessions, heart rate will be monitored using heart-rate monitors on the upper arm. All children, parents and therapists will come together during a 15-minute break for social interaction and a healthy snack to keep energy balance. After the break, the groups will switch activities. At the end of the session, all participants will reconvene to sum up the day.

#### Goal directed gross-motor training

Each session will include six to eight activity stations, tailored to the children´s ability and different goals. Each child`s goals will be displayed on the wall to make it easy for both therapists and families to remember the focus. The area will be equipped with materials such as mats, benches, trampolines, a small basketball hoop, a play kitchen, inviting toys and materials that arouse curiosity and promote interaction. Activities will address gross motor activities such as standing up, walking, turning, jumping, stairclimbing, or navigating short obstacle paths. Since the program is highly individualised, there will be specific instruction for each child on each station to support the child’s individually tailored training. The setup will aim to encourage high repetition practice and self-initiated movement through play. The therapists will provide guidance by modeling appropriate exercise techniques, offering feedback, and ensuring that the level of challenge is adjusted to the child’s individual capabilities and specific goals.

### The frame running training

To promote moderate- to high-intensity exercise, related to ability level and age, the room will be arranged with a straight track of 20 m allowing for variations in speed, duration and turning, while maintaining safety and motivation. Training will be individualized and tailored to each child’s abilities. Exercise intensity will be promoted through motivational, playful, game-based activities delivered by highly experienced therapists. These may include tag-style games with parents or therapists, searching for hidden objects, or collecting points. The space will feature thematic decorations and music to sustain engagement. Themes tailored to individual interests can be added to further enhance enjoyment and participation. All games will be structured to prompt spontaneous bursts of running, rapid directional changes, and acceleration. Specific adaptations to support participation across a range of abilities may include visual aids (e.g., color-coded pathways for children with visual impairments), acoustic adjustments for those with sound sensitivity, and additional verbal or visual cues to enhance comprehension and task execution. The aim is to achieve the highest possible intensity during the 30-minutes training session which will be monitored using heart rate monitoring.

#### Home training

Parents will be encouraged to continue daily practice at home using the Frame runner and activities aligned with their child’s goals, to promote repetition and consolidation. Treatment planning will be undertaken in collaboration with the family and will be guided by the GAS goals, with a focus on progressing to the next step. The plan will be reviewed with the therapist prior to the start of training and on a weekly basis; during each group training session, time will be allocated for families to discuss progress and any need for adjustments. The amount of training completed will be recorded in a weekly diary by parents.

#### Study protocol

Data collection will take place at the Astrid Lindgren Children’s hospital and at home according to the schedule shown in Tables 1 and 2; Fig. 1. The assessment takes about 1,5 h and the Gait analysis takes additional 1,5 h.

**Table 2:** Assessment to be collected at the different time points during the study, W = Week

Assessment	W 1	W 2	W3	W4	W5	W6	W7	W 8	W9	W10	W11	W 12
Prime SP	X			X				X				X
Accelerometry						X						
Heart rate	X	X	X	X	X	X	X	X	X	X	X	X
Diary	X	X	X	X	X	X	X	X	X	X	X	X

##### Quality assurance

Project organization will be managed by the PIs (ACE and FvW) and the study coordinator (AA). Data collection will be performed by a team of investigators, all previously experienced in collecting data from the actual assessments and not involved in the training session. Regular reviews of project progress and quality checks and quality assurance of all uploaded data will take place regularly on all data uploaded to the digital platform REDCap by one of the PIs (ACE) also responsible for exporting data when there is time for analysing data.

### Procedure for data collection

Data collection will take place during a two-hour session at the Astrid Lindgren Children´s hospital and gait analysis will take place on a separate occasion at the Motion analysis laboratory at Astrid Lindgren Children’s Hospital, Stockholm, Sweden. Additional data collection will occur during the intervention period via weekly logbooks and questionnaires regarding the children’s engagement in training, completed through REDCap. Physical activity intensity will also be measured using accelerometry and heart rate monitoring during the training sessions and at home. The complete assessment battery and timing across all study phases are summarized in Table 2. The assessment battery was selected to address the aims of the study as well as the hypothesis. The data collection will be administrated by the study coordinator (AA). The assessors have good knowledge and experience of performing the assessments in the clinic and for research. The observational assessments will be performed by an experienced pediatric physiotherapist (EH). Skeletal muscle morphology assessments (ultrasound) will be conducted by an expert in musculoskeletal imaging (MA) and a pediatric radiologist (OK). Activity monitoring and physiological measurements during the intervention (accelerometry, heart rate monitoring) will be supervised by a medical doctor (JS) and an exercise physiologist (JN). Gait analysis will be performed at the Motion analysis laboratory by a team of lab staff and clinicians (CL, AE, KA). The study coordinator (AA) will collect questionaries and support parents to formulate individually tailored goals for each child prior to the start of the intervention. Weekly home-based training diaries and engagement questionnaires will be completed in REDCap by parents throughout the intervention period (Table 1). The different assessments that will be used and at what time points data will be collected are described in Table 1.

### Primary outcome measure

#### Gross motor capacity

*Gross Motor Function Measure (GMFM-66)*, is an observational, standardized and criterion-referenced measure, developed to evaluate change in gross motor function in children with cerebral palsy [45]. The 66 items cover gross motor capacity from lying and rolling, to walking, running and jumping. Item scores (0–3) are converted to an interval score from 0 (low capacity) to 100 (high capacity) (Rasch analysis), where typically developing five-year-olds are expected to achieve a score of 100. GMFM-66 is reported to be valid, reliable and responsive to changes in gross motor capacity in children with CP [46]. In addition, the subscales “D. Standing and E. Walking and Jumping will be used from GMFM-88 [47]. The assessments will be performed barefoot and video-recorded to allow verification of scoring and reliability check if needed. To assess inter-rater reliability and strengthen the internal validity of the study, a subset of the GMFM recordings will undergo blinded secondary scoring (see statistics).

### Accelerometer set-up and analysis

The SENS Motion system is a thigh-worn accelerometer measuring 3-axis acceleration. Accelerometry data will be collected at five different time points during the study period (Tables 2 and 1). The raw acceleration data will be compared between time points as a measure of total activity and time presented as non-sedentary activity (minutes). Data will also be analyzed based on individual cut-off values (percentages of max acceleration during 30 m Frame Running sprint) and presented as minutes/day in different intensity zones. Accelerometers will be attached with adhesive tape to the outer thigh of the dominant/least affected leg. They will be worn for seven consecutive days, including an entire weekend. In a diary, parents note any unusual physical activity or inactivity, such as bed rest due to illness, to inform subsequent data analysis.

### Secondary outcomes

#### Classifications and standardized assessments

*The Gross Motor Function Classification System – Expanded and revised (GMFCS-ER) and the Manual Ability Classification System (MACS)* are five-level classification systems describing gross motor function and manual ability in children with CP. The appropriate level is determined after discussions with caregivers and therapists who are familiar with the definitions of each level [48, 49].

*Pediatric Evaluation of the Disability Inventory (PEDI-cat)* is a norm and criterion-referenced questionnaire which evaluates the child’s daily performance and need of caregiver assistance. In this study we will use the long version of mobility [50]. A structured interview with the parents will be conducted to obtain information about the child’s performance in the daily environment. The summary scores can be converted to normative standard scores and scaled scores. The normative scores are available for children 6 month to 7.5 years. The scaled scores range on a continuum from 0 to 100. PEDI is available in a Swedish version [51].

*Functional Mobility Scale (FMS)* describes mobility performance across three distances − 5 m, 50 m, 500 m, and the child’s use of assistive devices, in daily life. It determines the level of independence and need for assistive devices [52]. The assessment is based on a brief interview rather than direct observation. The clinician asks how the child usually moves around short distances indoors (5 m, e.g., at home), medium distances (50 m e.g., in and between rooms at preschool), and long distances (500 m, e.g., in a shopping center). Ratings reflect what the child does (performance), not capacity.

#### Walking, running and Frame Running tests

Three capacity time tests (10-m walk/run, Timed Up and Go, and 30-m Frame Running) will be conducted under standardized and comparable conditions. For children who normally use walking devices or orthoses, the child and parent will select the preferred device together and decide whether orthoses should be worn during testing. All tests will be performed with a stopwatch using the same protocol: three trials will be completed, and if the third trial is the fastest and the child is able to continue, a fourth trial will be added (maximum four trials). All trials will be video recorded to enable subsequent evaluation of gait characteristics such as number of steps, stride pattern, and movement quality. In addition, test conditions—such as the environment, motivational cues, and any unexpected events will be documented to ensure reproducibility across all assessment occasions.

*Time up and go* (TUG) is an observational assessment. TUG measures the time (in seconds) required for an individual to stand up from a standard chair, walk 3 m, turn around, walk back to the chair, and sit down again. In this study, children will start from a height-adjusted stool that allowed sitting position of approximately 90° at the hip and knees. A tape mark on the floor indicates the 3-meter turning point. The test has been widely used in clinical practice as an outcome measure to evaluate functional mobility and dynamic balance [53].

*Time test 10-meter walk/run*, means that the child walks or runs 10 m at their fastest speed.

*Time test 30 m Frame Running* means that the child completes a 30 m straight-line time trial at maximal speed using an individually fitted FR.

### Three-dimensional gait analysis

A three-dimensional twelve-camera motion analysis system (3DGA) (Vicon-UK^*Ⓡ*^, Oxford, UK) including synchronized video recordings with two digital cameras and two force plates (Kistler^*Ⓡ*^, Winterthur, Switzerland) embedded in the floor at the Motion analysis Laboratory at Astrid Lindgrens Children’s Hospital, Stockholm, Sweden is used to describe gait. With the system objective information such as pelvis, hip, knee and ankle - angles (kinematics) and forces (kinetics), and spatiotemporal parameters including step length and walking speed throughout the gait cycle are provided [54]. Ground reaction forces on the joints (kinetics) are measured from two force plates (Kistler^*Ⓡ*^, Winterthur, Switzerland) embedded in the floor. A lower limb 16 markers (10 mm in diameters) biomechanical model (plug-In-Gait). A two-dimensional video vector system can be applied giving information on direction of the ground reaction forces on the joints in case of no acceptance by the child for markers to be put on the skin. 3DGA is frequently used to describe gait, and to evaluate the effect of interventions on gait of children with CP [55, 56]. Prior to recordings, anthropometric measurements such as height, weight, and the width of the body segments will be collected before markers are placed on anatomical landmarks on the child’s body. During testing children will walk (i) barefoot, and (ii) with orthoses and/or habitual shoes on a 8 m walkway at a self-selected walking speed until three trials are obtained.

*Passive range of motion*: A physical examination including bilateral measurements of passive range of motion (pROM) of hip (flexion, extension, abduction), knee (flexion, extension, hamstrings angle), and ankle (dorsiflexion with flexed and extended knee) with a goniometer will be performed [57].

*The Modified Ashworth Scale* will be used to assess spasticity of muscles around hip, knee, and ankle. This is an ordinal scale (0, 1,+ 1 2, 3, 4) measuring resistance to passive elongation of a muscle at rest [58]. Furthermore, throughout the testing dyskinesia will be assessed as present or not (yes/no), and if present assessed as dystonia and/or chorea-athetosis (yes/no) [59]. Additionally, presence of ataxia will be observed and registered as present or not (yes/no). All examinations will be performed by experienced pediatric physiotherapists (CL, AE).

### Skeletal muscle and subcutaneous fat thickness

Objective assessment of muscle morphology will be performed using non-invasive, pain-free ultrasound imaging on three occasions over the five-month intervention period. B-mode ultrasound will be utilized to quantitatively measure skeletal muscle and subcutaneous fat thickness (in millimeters) and qualitatively assess muscle architecture (shape and grey scale analysis). Specifically, muscle thickness is measured for the thigh (encompassing the vastus lateralis and vastus intermedius muscles collectively) and the calf (medial gastrocnemius muscle) in both lower extremities at predefined positions based on anatomical landmarks, as detailed in prior publications. Measurements will be performed using a stationary ultrasound system (Canon Aplio A; Canon medical systems corporation, Tochigi, Japan).

### Goal Attainment Scaling (GAS)

The GAS is an individualized, criterion-referenced outcome measure that is sensitive to change and well established within pediatric rehabilitation [36, 60]. The procedure involves describing the baseline level of performance and specifying a range of possible outcomes for each goal. The scale comprises five levels, ranging from −2 to +2, where −2 represents baseline performance, 0: expected level of goal achievement and +2: performance substantially exceeding the expected goal. Ratings of goal attainment are aggregated and converted into an overall T-score. A T-score of 50 represents achievement of the expected goal (level 0) and denotes a clinically meaningful improvement. T-scores derived post-intervention and at follow-up were used for analysis. Parents will video-record the goals, before and after the intervention period.

### Heart rate measures

Heart rate monitoring (Polar, Kempele, Finland) provides an objective measure for describing the intensity of physical activity. These are non-invasive and are worn with a band around the upper arm or the wrist. The children wear the heart rate monitors during the training sessions, and the accelerometers at home to estimate daily physical activity.

### Questionnaires

Prime-P: The Pediatric Rehabilitation Intervention Measure of Engagement – Parent version (PRIME-P) [61] is a self-report measure. The questions are about what parents thought, how they felt, and what they did during their child’s therapy session and how the session has affected their overall feelings about the intervention process.

PrimeSP: The Pediatric Rehabilitation Intervention Measure of Engagement - Service Provider version (PRIME-SP) captures therapists´ observations of client engagement in an intervention session, with engagement defined as active involvement and investment in the client role [62]. The PRIME-SP has three parts - an overall rating of engagement, ratings of aspects of engagement (affective, cognitive, and behavioural involvement), and a place to record observations of factors that may have influenced client engagement.

An electronic diary: To ensure sufficient training intensity, parents were asked to document the daily duration of goal-related practice and Frame running. An electronic form will be sent out once a week during the training period. Parents will be asked to summarize the training time and answer a few questions about feasibility.

### Evaluation of the treatment

Following completion of the study period, participants will be invited to complete an electronic evaluation questionnaire. The questionnaire comprises 20 questions assessing participants’ experiences of study participation, perceptions of the project organization, the feasibility of conducting training both at home and in group-based sessions, and the adequacy of the support provided.

### Fidelity

Intervention fidelity will be examined at several time points during the trial to assess how closely the *Full Speed Ahead* program is delivered according to its intended design. Fidelity will be described in terms of adherence, dose, quality of delivery, and participant engagement [63].

Adherence to the intervention protocol and training dose will be monitored using attendance records from group sessions and weekly training diaries completed by parents. Quality of delivery will be evaluated using the PRIME-O, which captures therapists’ engagement, attitudes, and use of coaching strategies during the intervention. Child engagement will be assessed using the PRIME-P, in which parents rate their child’s engagement; these measures will be collected repeatedly during both the assessment and training phases. In addition, therapists will rate engagement during group training sessions by PRIME-SP. Physiological measures, including heart rate monitoring and accelerometer data, are outcome measures but will also be used as complementary indicators of fidelity, reflecting the intensity and amount of physical activity delivered.

### Safety and ethics

Frame Running can be performed at high speeds and therefore carries a risk of falls and collisions with other people or surrounding objects. An information sheet on risk factors has been prepared and will be given to the families. To mitigate these risks, children will be required to wear a helmet during Frame Running, the training area will be kept clear of obstacles not included in the program, and a designated adult (parent and/or therapist) will always remain close to the child. In addition, because the intervention aims to promote moderate- to high-intensity exercise, there is a potential risk of fatigue and overexertion, particularly in children who are not accustomed to vigorous activity. Trainers will therefore monitor the child’s tolerance during each session, adapt the intensity, provide rest breaks when needed, and stop the activity if safety concerns arise. Participation is voluntary, and the child’s interests will be respected throughout. As young children may have limited ability to articulate discomfort, therapists will actively attend to behavioural cues (e.g., distress, withdrawal, pain behaviour, or marked fatigue and will pause or discontinue the session if the child does not wish to continue. The intervention is intended to be challenging yet enjoyable, and children will never be pressured to participate beyond their comfort or capability. All adverse events and other unexpected incidents will be documented throughout the study in accordance with a predefined adverse events procedure and reported as required, and parents will receive written safety information before the intervention starts.

## Discussion

The FSA program aims to address the need for effective interventions targeting gross motor function and health in young children with CP. Currently, there is a lack of evidence for effective training programs for this group of children [11, 64]. In particular, research evaluating interventions for non-ambulant children with CP is scarce; most existing studies have focused on children classified as GMFCS levels I to III, leaving those at levels IV and V underrepresented and often without access to evidence-based interventions [65].

By implementing a new early and intensive program, consisting of a combination of Frame Running and goal-directed training, the FSA program aims to facilitate higher levels of functioning and participation than would be expected without such intervention, both short and long term. If proven effective, this approach could have substantial implications for planning both short- and long-term treatment and support services for young children with CP.

The prospective repeated-measures intervention design allows detailed analysis of individual response patterns over time, while also supporting group-level analyses. This is an advantage, as the variation among children is considerable and prospective repeated-measures intervention design allows for a broad understanding of the intervention impact on each participant, supporting the development of more effective and personalized treatment strategies for children with CP.

This study has limitations. The prospective repeated-measures intervention design does not include randomization or a control group, which limits causal inference. Blinding is also limited, as participants and therapists cannot be blinded to the intervention, although independent assessors will perform outcome assessments whenever possible. In addition, the relatively small sample size and the heterogeneity of children with CP may limit generalizability. Variation in adherence to home-based training may also affect outcomes. However, repeated assessments across study phases and the combination of individual- and group-level analyses are expected to provide important information about the feasibility and potential effects of the intervention.

Despite these limitations, the study is expected to provide important knowledge about the feasibility and potential effects of an early, intensive, family-centered mobility intervention for young children with CP. The findings may help guide future intervention development and inform clinical practice for this underserved question.

## Data Availability

No datasets were generated or analysed during the current study.

## Abbreviations

CP: Cerebral palsy
FSA: Full Speed Ahead
pROM: passive range of motion
GAS: Goal Attainment Scaling
